# New Appliances and Things Medical

**Published:** 1898-06-11

**Authors:** 


### NEW APPLIANCES AND THINGS MEDICAL.

[We shall be glad to receive, at our Office, 28 & 29, Southampton Street, Strand, London, W.C., from the manufacturers, specimens of all new preparations and appliances which may be brought out from time to time.]

### MEDICATED SOAPS AND BATH POWDERS.

(Charles Midgley, Limited, 22, St. Ann's Square, Manchester.)

The method of applying medicaments to the skin by means of soap is rapidly coming into general favour, and undoubtedly in many cases it is justifiable, replacing the clumsy and inelegant method of treating the skin by ointments. Soaps, however, in the form of cakes do not invariably form the best bases for applying drugs in definite proportion or of definite constitution, for the character of the drug may become changed or lost during the manipulation which is necessary for its preparation. The above firm have therefore adopted the method of incorporating a number of dermatological medicaments with powdered soap, either super-fatted or alkaline. Medicated baths can thus be enjoyed by the public without special and elaborate preparation. All that is necessary is for the dermatologist in charge to choose the drug and specify the strength. Messrs. Midgley will, to his order, supply the medicated powdered soap, which is then used in the ordinary way. We recommend Messrs. Midgley's specialities to the notice of all dermatologists.

### ENGLISH AND FRENCH MUSTARDS.

(Sadler, Firth, and Ross, Great Guildford Street, Southwark, London.)

We have received samples of each of the above preparations. We find them both of good quality, carefully prepared, and free from impurities. The French mustard we consider a particularly elegant condiment, with highly piquant and agreeable flavouring. Being of British manufacture it has additional claims to our notice. We can recommend both examples of Sadler's speciality to the notice of our readers as excellent and pure specimens of flour of mustard and prepared French mustard respectively.

### DISINFECTANT SOAP.

(Sanitas Company, Limited, Letchford's Buildings, Bethnal Green, London, E.)

This soap is, as it claims to be, a truly disinfectant soap. It has an agreeable and particularly fresh odour, and for scrubbing floors of bedrooms, dormitories, kennels, &c., it has all the necessary quality both as regards antiseptic and cleansing qualities. It forms a good lather and constitutes a fine deodorant. The only criticism that we can suggest after a somewhat extended trial is that the soap is apparently too new—it contains rather a high percentage of water and somewhat readily breaks. These are faults which time will naturally remedy.

### BUCHANAN'S SCOTCH FOOD.

(Buchanan, Sons, and Foster, 275, Strand, London, W.C.)

This highly nutritious food has been submitted to our notice as a speciality for infants and invalids. The food is easily prepared by mixing with water and milk, and then boiling for a few minutes. The resulting mixture is highly agreeable, readily digested, and particularly rich in those bone and flesh forming constituents which are essential for rapidly-growing children and convalescents who have lost weight in wasting diseases. Buchanan's Scotch Food should be given with the greatest caution to infants under six months of age, and then only as a supplement to, and not as a substitute of, human or cows' milk. From that age onwards in our opinion its value is far greater, and for invalids and aged persons it is a food of great flesh-forming qualities.

### FROMM'S SOUP TABLETS.

(Fromm's Extract Company, Ltd., 5, White Street, London, E.C.)

We have recently received samples of desiccated soup from Fromm's Extract Company. The sample submitted to us is a green vegetable soup, supplied in the form of a disc about three and a-half inches in diameter, and half an inch thick, securely done up in specially-prepared paper. The form of the tablets is most convenient for storage purposes, and as far as we are able to judge, after keeping the preparation for about three months, it has suffered no deterioration in quality or flavour. Each tablet costs 2½d., and when prepared according to the directions, makes a most agreeable soup sufficient for about nine persons.

### NEW GLASS TANK FOR SURGICAL DRESSINGS.

(Reynolds and Branson, 13, Briggate, Leeds.)

These tanks are made entirely of glass except the lids, which are of metal. They are strong and convenient in shape, and the lid fits accurately and tightly; they are intended for keeping surgical dressings in a cleanly and aseptic condition. For surgical practice in town or country they are of great value, as the contents can be seen at a glance without removing the lid or sorting out the various dressings. We have made constant use of one of the smaller-sized tanks for carrying dressings ordinarily employed in practice, and have found t answer all the requirements of the case.

